# Anthracycline-based chemotherapy in extraskeletal myxoid chondrosarcoma: a retrospective study

**DOI:** 10.1186/2045-3329-3-16

**Published:** 2013-12-18

**Authors:** Silvia Stacchiotti, Gian Paolo Dagrada, Roberta Sanfilippo, Tiziana Negri, Isabella Vittimberga, Stefano Ferrari, Federica Grosso, Gaetano Apice, Marco Tricomi, Chiara Colombo, Alessandro Gronchi, Angelo P Dei Tos, Silvana Pilotti, Paolo G Casali

**Affiliations:** 1 Adult Mesenchymal Tumor Medical Oncology Unit, Cancer Medicine Department, Fondazione IRCCS Istituto Nazionale Tumori, Milan, Italy; 2 Experimental Molecular Pathology Unit, Department of Pathology, Fondazione IRCCS Istituto Nazionale Tumori, Milan, Italy; 3 Cancer Medicine, Oncologia Medica Azienda Ospedaliera della Provincia di Lecco, Lecco, Italy; 4 Chemotherapy Department, Istituto Ortopedico Rizzoli, Bologna, Italy; 5 Oncology, SS Antonio e Biagio General Hospital, Alessandria, Italy; 6 Sarcoma Medical Oncology Unit, Istituto Nazionale Tumori, Fondazione Pascale, Naples, Italy; 7 Rare Cancer Network, Fondazione IRCCS Istituto Nazionale Tumori, Milan, Italy; 8 Department of Surgery, Fondazione IRCCS Istituto Nazionale Tumori, Milan, Italy; 9 Department of Anatomic Pathology, General Hospital of Treviso, Treviso, Italy

**Keywords:** Sarcoma, Chondrosarcoma, Extraskeletal myxoid chondrosarcoma, Chemotherapy, Anthracycline, Doxorubicin, Ifosfamide

## Abstract

**Background:**

Extraskeletal myxoid chondrosarcoma (EMC) is a rare subgroup within soft tissue sarcomas. Its sensitivity to chemotherapy is reported to be low.

**Methods:**

We retrospectively reviewed a series of 11 EMC patients treated as from 2001 within the Italian Rare Cancer Network (RCN) with anthracycline-based chemotherapy. Pathologic diagnosis was centrally reviewed in all cases and confirmed by the presence of the specific chromosomal rearrangements, involving the *NR4A3* gene locus on chromosome 9.

**Results:**

Eleven patients treated with anthracycline-based chemotherapy were included (M/F: 9/2 – mean age: 52 years – site of primary: lower limb/other = 9/2 - metastatic = 11 – front line/ further line = 10/1 – anthracycline as single agent/ combined with ifosfamide = 1/10). Ten patients are evaluable for response. Overall, best response according to RECIST was: partial response (PR) = 4 (40 %), stable disease (SD) = 3, progressive disease (PD) = 3 cases. Median PFS was 8 (range 2–10) months.

**Conclusions:**

By contrast to what reported so far, anthracycline-based chemotherapy is active in a distinct proportion of EMC patients.

## Background

Extraskeletal myxoid chondrosarcoma (EMC) is a very rare sarcoma of uncertain differentiation [1] that, despite its name, does not exhibit any cartilaginous differentiation. EMC usually originates from the deep soft tissue, the thigh being the most common site [2]. Demicco and Coll. recently reported on 5 cases of molecularly confirmed EMC arising primarily in the bone [3]. On this basis, they proposed to relabel this tumor as myxochondroid sarcoma, either osseous or extraskeletal.

Microscopically, EMC can be subdivided into a conventional well-differentiated and a cellular high-grade EMC, the latter being marked by the presence of a predominantly epithelioid morphology, high mitotic rate and necrosis [1]. Cases of dedifferentiated ECM were also described [4].

EMC is marked by a specific chromosomal rearrangements, involving the *NR4A3* gene locus on chromosome 9 [1,5]. More often *NR4A3* (also called *CHN* or *TEC*) is fused to *EWSR* on chromosome 22 [6,7], although four chimeric variants were described to date [4,8,9]. *NR4A3* translocation is relevant in case of differential diagnosis with other myxoid-mesenchymal neoplasms [10].

The natural history of the EMC is marked by a relatively indolent behavior with a 10-year survival rate ranging between 65% and 85%, and 40% risk of metastases at 10 years [11,12], lung being the most frequent site of secondary lesions.

Available literature reports EMC as a disease poorly sensitive to cytotoxic chemotherapy [11-15].

We herein report on a retrospective series of 11 patients with locally advanced/metastatic EMC, molecularly confirmed by the presence of *NR4A3* rearrangement*,* treated with anthracycline-based chemotherapy at our institution and within the Italian Rare Cancer Network.

## Methods

### Patients selection

We retrospectively identified 11 patients with progressive, metastatic, molecularly confirmed EMC treated with anthracycline-based chemotherapy at Fondazione IRCCS Istituto Nazionale Tumori, Milano and those included in the data-base of the Italian Rare Cancer Network, registered by other Italian institutions, from January 2001 to June 2013. The analysis was approved by the Institutional Ethics Committee.

Pathological diagnosis was centrally reviewed in all cases by 2 expert pathologists (SP and APDT) and confirmed by the evidence of *NR4A3* rearrangement. All patients had evidence of Eastern Cooperative Oncology Group performance status (ECOG PS) ≤3 and an adequate bone marrow and organ function. All patients provided a written informed consent to data collection within the network and to the treatment. Data were extracted from individual patient file and analyzed.

### Pathology and molecular analysis

The diagnosis was rendered according to the last WHO classification [1]. Immunoprofile assessment was performed using the antibodies and conditions detailed in Table 1.

**Table 1:** Immunohistochemistry conditions

**Antibody**	**Clone**	**Company**	**Dilution**	**Antigen retrieval**
S100	Polyclonal	Dako	1′:4000	citrate buffer, 15′
Synaptophysin	DAK-SYNAP	Dako	1′:200	EDTA, 30′
EMA	E29	Dako	1′:250	EDTA, 30′
PPARγ	E-8	Santa Cruz Biotechnology	1′:20	EDTA, 30′

FISH was carried out on FFPE tissue samples with commercially available *EWS* break apart probe (VYSIS LSI-EWSR1 dual color break apart) and with two *NR4A3* specific BAC probes (obtained from C.H.O.R.I. BAC PAC resources): Spectrum Orange labeled RP11-30 L7 for the 5′ end and Spectrum Green labeled RP11-30 N20 for the 3′ end of the gene. Probe labeling and FISH procedure were carried out as previously described [16]. Cases with a morphology consistent with EMC but without the evidence of *NR4A2* rearrangement were excluded from this series.

### Treatment

Patients were treated with anthracyclines as single-agent (doxorubicin 60–75 mg/smq, i.v., bolus), or in combination with ifosfamide (epirubicin 105 mg/smq + ifosfamide 9000 mg/smq, i.v., in 3 days). Mesna was added to ifosfamide. Chemotherapy was administered every 3 weeks, together with steroids and antiemetics. Prophylactic granulocyte colony stimulating factors were administered.

Treatment was withheld for haematologic grade ≥3 adverse events (AE) and for non haematologic grade ≥2 AE (as defined by the National Cancer Institute Common Toxicity Criteria, version 3.0) and restarted after recovery to grade <2 in case of haematologic or grade <1 in case of non-haematologic.

### Clinical assessment

Full blood cell count and biochemistry were assessed at baseline and before every administration. AE were recorded. Disease status was assessed at baseline by a whole body computed tomography scan (CT), a CT or magnetic resonance (MRI) of the site(s) of disease, and a whole body bone scan. CT/MRI were repeated after the first 2 or 3 cycles of treatment then every 2–3 months.

Response to treatment was evaluated by Response Evaluation Criteria in Solid Tumor (RECIST) [17].

### Statistical analysis

Progression-free survival (PFS) and overall survival (OS) were estimated with Kaplan-Meyer method [18]. Failure for PFS was progressive disease according to RECIST, or death. OS, failure was death due to any cause. For PFS and OS, patients who interrupted their treatment without evidence of disease progression and underwent complete surgery were censored at the time of surgery. Alive patients were censored at the time of the last contact.

## Results

Eleven patients with measurable disease were treated with an anthracycline-based chemotherapy. Five patients with a previous diagnosis of EMC were not included in this series since diagnosis was not confirmed by the presence of *NR4A3* rearrangement. Ten patients were evaluable for response (in one case treatment was interrupted early due to toxicity). Main patient characteristics were: male/female 9/2, mean age 52 years, primary arising from soft tissue/bone 10/1, locally advanced/metastatic 1/10 (lung metastases in 10 cases), ECOG PS ≤2: 11, anthracycline-based chemotherapy as front-line/further line: 10/1. Three patients underwent macroscopic complete surgery after treatment. Patient characteristics are detailed in Table 2.

**Table 2:** Patient clinical characteristics and response evaluation

**Patient ID**	**Gender**	**Age at time of chemotherapy (years)**	**Diagnosis**	**NR4A3 rearrangement**	**Site of primary tumor**	**Staging at time of initial diagnosis**	**Site of relapse at the time of chemotherapy**	**RECIST evaluation**	**PFS**
1	F	48	EMC	yes	thigh	localized disease	abdomen, LN	PR	7*
2	M	56	EMC	yes	thigh	localized disease	lung, LN	PD	4
3	M	46	EMC	yes	thigh	local + lung	lung, LN	PR	8
4	M	38	EMC	yes	leg	localized disease	lung, bone, liver, soft tissue	NV	2
5	M	55	EMC	yes	leg	localized disease	lung, LN, soft tissue	PR	8
6	F	64	EMC	yes	thigh	localized disease	lung	SD	10
7	M	69	EMC	yes	buttock	local + lung	buttock, lung	PR	5*
8	M	55	EMC	yes	arm	local + lung	lung, LN	SD	7
9	M	51	EMC	yes	thigh	localized disease	lung	PD	4
10	M	52	EMC	yes	leg	localized disease	lung	SD	5*
11	M	48	EMC	yes	sacrum	local + lung	lung	PD	3

*Patient treated with surgery after chemotherapy, censored at the time of surgical resection; *M*, male; *F*, female; *EMC*, extraskeletal myxoid chondrosarcoma; *LN*, lymphonode; *PR*, partial response; *SD*, stable disease; *PD,* progressive disease; *NV*, not evaluable; *PFS*, progression free survival.

### Treatment

The median number of cycles of chemotherapy was 4 (range: 1–8). Patients received anthracycline as single agent in 1 case, in combination with ifosfamide in 10. One patient stopped his treatment after the first cycle due to toxicity.

Overall, toxicity was as expected, with ≥G2 neutropenia in 60% of patients, and nausea, vomiting, asthenia, fever and mucositis as the main non-haematologic toxicities.

### Pathology and molecular analysis

Immunophenotypic analysis is detailed in Table 3.

**Table 3:** Immunohistochemistry and FISH results

**Pt ID**	**S100**	**Synaptophysin**	**EMA**	**PPARγ**
1	-	-	+ strong, diffuse	+
2	-	-	+ plurifocal	+
3	-	-	-	+ weak
4	-	+	+	+
5	-	-	-	+
6	-	-	-	+
7	+	not done	+	+
8	-	-	+	+
9	+ focal	-	+ focal	+
10	-	-	+	-
11	+	not done	-	not done

All tumors included in this series were positive for *NR4A3* rearrangement. *EWS* was rearranged in 9 of 11 cases, as shown in Table 3. Five more patients with a previous diagnosis of EMC were eventually excluded from this series since diagnosis was not confirmed by the presence of *NR4A3* rearrangement.

### Response

**Figure 1:**
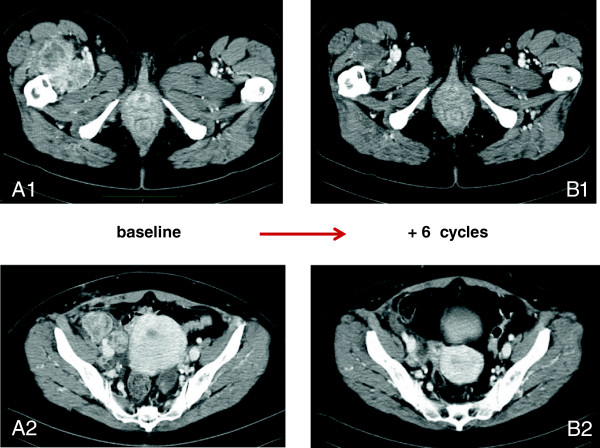
**Response to chemotherapy with epirubicin and ifosfamide.** CT scan (arterial phase after contrast medium). Thigh primary extraskeletal myxoid chondrosarcoma (**Panel A1**) with concomitant intra-abdominal lymphonodes involvement (**Panel A2**) at baseline, and after 6 cycles of treatment with epirubicin and ifosfamide (**Panel B1 and B2**, respectively). The response is marked by a >30% decrease in tumor size thus classifying for a partial response by RECIST.

Ten patients were assessable for response, as detailed in Table 2. The best response according to RECIST was: partial response (PR) in 4/10 cases (40%), stable disease (SD) in 3/10 (30%), with a minor response in one of them. Progressive disease (PD) was observed in 3/10 (30%) cases. Disease control rate was 70%. Responses were confirmed at 3 months. Responses were observed in 4 patients treated with epirubicin plus ifosfamide and in one patient treated with doxorubicin as a single agent. Figure 1 shows a response to doxorubicin as single agent in a previously progressive patient.

**Figure 2:**
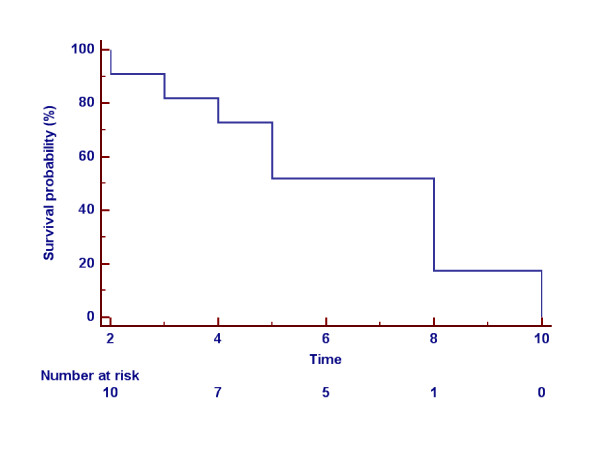
**Overall progression free survival (PFS) of patients treated with anthracycline-based chemotherapy*.*** Median PFS 8 months*.*

Median OS was 30 months (range 10 mos-13 years). At a median follow-up of 30 months, the estimated OS at 10-year was 50%, with 2 patients dead at the time of the present analysis and one lost to follow-up. The median PFS for the entire group was 8 months (range 2–10), with 50% patients progression-free at 6 months (Figure 2). Three patients (patient 1/7/10, Table 2) underwent complete surgical resection after chemotherapy, with evidence of a new distant relapse after 24/12/24 months from surgery, respectively.

## Discussion

We retrospectively analyzed 11 patients with progressing, advanced, molecularly confirmed EMC, treated with anthracycline-based chemotherapy since 2001 within the Italian Rare Cancer Network. We observed 4 RECIST PR out of 10 patients evaluable for response, with a median PFS of 8 months and 50% patients progression-free at 6 months. As EMC is an extremely rare mesenchymal malignancy, rare cancer networks represent a valuable tool in order to collect case series, in addition to sharing and developing clinical expertise.

In fact, EMC represents a small subgroup among sarcomas and no prospective study focusing on their medical treatment is available as of today. Despite rarity, in order to collect an homogeneous series, we decided to include only cases in which diagnosis had been confirmed by the presence of *NR4A3* rearrangement.

In our series the response rate to anthracycline-based chemotherapy looks greater than previously reported. The only responses to chemotherapy were described in 2001 by McGrory in 2 of 6 metastatic EMC patients responsive to a multi-agent chemotherapy [13], and more recently by Han who observed a complete remission in one patient treated with anthracyclines plus ifosfamide [19]. No objective responses were observed in the two largest retrospective series published so far, which collected cases selected over a period of 30 years starting from the 70′s [11,13]. In fact, no patient had a response amongst 10 treated with doxorubicin and dacarbazine-based regimens in the MD Anderson’s retrospective study [13] published in 1995, as well as none of the 21 patients treated with different regimens, mostly anthracycline-based, reported by Memorial Sloan Kettering Cancer Center and Royal Marsden Hospital in 2008 [11]. In the latter analysis, the best response was represented by stable disease lasting ≥ 6 months in only 25% of cases, with an estimated 40% median-PFS at 6 months. More recently, Ogura and Coll. reviewed their institutional series of 22 patients, with no response in 4 cases treated with ifosfamide-based chemotherapy [12]. An explanation for this discrepancy may well be that diagnostic criteria for EMC have improved in the last years and possibly older series may have included other histological types with overlapping morphologies, such as myoepithelial carcinomas. In particular, the analysis to detect *NR3A4* translocation, which is specific of EMC, was described for the first time in 1985 [5] and was not routinely used to confirm the diagnosis until recently. It is now evident that EMC are morphologically and molecularly distinct from conventional bone chondrosarcoma, whose lack of sensitivity to chemotherapy is well known [20,21]. Indeed, EMC and conventional bone chondrosarcomas are completely unrelated.

As already mentioned, the differential diagnosis is rather broad and includes malignant myoepithelioma/myoepithelial carcinoma, whose natural history and chemosensitivity is still not well understood [1,22], but also low grade fibromyxoid sarcoma, myxoid liposarcoma and synovial sarcoma with myxoid changes [22-24]. Immunohistochemical analysis plays an important role: however, there is some degree of overlapping that may represent an additional challenge [1,24]. Although not frequently observed, synaptophysin expression [25] has been recently confirmed by gene expression profiling analysis [23]. Finally, PPR-gamma, firstly described to be involved in ECM signaling pathway [23], is also expressed in many other cancers including myxoid liposarcomas [26-28], and therefore it can hardly be diagnostically helpful. From these findings it appears that cytogenetic-molecular analysis substantially helps in distinguishing EMC from other tumor entities. For these reason, due to the lack of *NR4A3* rearrangement, we excluded 5 of 16 cases initially diagnosed as EMCS according to morphology/immunohistochemistry.

EMCS is an indolent disease and in some cases it is characterized by a slow progression also in the metastatic phase, affecting about 40% of cases. However, in case of advanced and progressive disease, a medical treatment is needed. Our series suggests that anthracycline-based chemotherapy can have a role in this setting. Of note, 3 patients of our series were completely resected after having a response to chemotherapy. None of them was cured, but they all recurred after >12 months from surgery, suggesting that chemotherapy may have played a role.

In case of resistance to conventional cytotoxic chemotherapy, further medical treatment are needed in EMC. We recently reported on the activity of sunitinib in two patients carrying a metastatic EMC pretreated with chemotherapy [29]. These preliminary results are under confirmation in a larger series, while a prospective study on pazopanib, another antiangiogenic agent, is planned.

## Conclusions

By contrast to what reported so far, anthracycline-based chemotherapy is active in a distinct proportion of EMC patients. Series like ours may serve as external controls for future clinical studies on new agents in such a rare histology.

## Competing interests

None of the authors declared any financial or non-financial competing interests.

## Authors’ contributions

SS contributed to study design and coordination, data collection, analysis and interpretation, and drafted the manuscript. RS, IV, SF, FG, GA, MT, CC, AG contributed to data collection and interpretation, and drafting. TN, GD, APD and SP carried out the molecular genetic studies and the pathologic review, and drafted the manuscript. PGC contributed to data analysis and interpretation, and drafted the manuscript. All authors read and approved the final manuscript.
